# Investigation on the Conductive Filament Growth Dynamics in Resistive Switching Memory via a Universal Monte Carlo Simulator

**DOI:** 10.1038/s41598-017-11165-5

**Published:** 2017-09-11

**Authors:** Yu Li, Meiyun Zhang, Shibing Long, Jiao Teng, Qi Liu, Hangbing Lv, Enrique Miranda, Jordi Suñé, Ming Liu

**Affiliations:** 1 Key Laboratory of Microelectronics Devices & Integrated Technology, Institute of Microelectronics of Chinese Academy of Sciences, Beijing, 100029 China; 2 University of Science and Technology Beijing, Beijing, 100083 China; 3 Departament d’Enginyeria Electrònica, Universitat Autònoma de Barcelona, Bellaterra, 08193 Spain; 4 University of Chinese Academy of Sciences, Beijing, 100049 China; 5 Jiangsu National Synergetic Innovation Center for Advanced Materials (SICAM), Nanjing, 210023 China

## Abstract

In resistive random access memories, modeling conductive filament growing dynamics is important to understand the switching mechanism and variability. In this paper, a universal Monte Carlo simulator is developed based on a cell switching model and a tunneling-based transport model. Driven by external electric field, the growing process of the nanoscale filament occurring in the gap region is actually dominated by cells’ conductive/insulating switching, modeled through a phenomenological physics-based probability function. The electric transport through the partially formed CF is considered as current tunneling in the framework of the Quantum Point Contact model, and the potential barrier is modulated during cells’ evolution. To demonstrate the validity and universality of our simulator, various operation schemes are simulated, with the simulated *I* − *V* characteristics well explaining experimental observations. Furthermore, the statistical analyses of simulation results in terms of Weibull distribution and conductance evolution also nicely track previous experimental results. Representing a simulation scale that links atomic-scale simulations to compact modeling, our simulator has the advantage of being much faster comparing with other atomic-scale models. Meanwhile, our simulator shows good universality since it can be applied to various operation signals, and also to different electrodes and dielectric layers dominated by different switching mechanisms.

## Introduction

Since the resistive switching (RS) effect induced by electric stimuli was first discovered by Simmons and Verderber in 1967^1^, much research efforts have been made to understand the underlying switching mechanism and many materials have been considered for the development of RS devices, such as resistive memories (RRAM) and threshold switching devices^2–4^. Comparing to existing charge-based flash memory, RRAM, which has a very simple three-layer sandwich structure, has many advantages, in terms of fast switching speed (down to ~10 ns^5^), high integration density (scaling down to ~10 nm × 10 nm in each unit^6^) and low power consumption (with sub-picojoule switching per bit^7^). By virtue of these advantages, RRAM is considered as one of the main candidates for next generation non-volatile memories by the International Technology Roadmap for Semiconductors (ITRS)^8^. However, the reliability, stability, and uniformity of RRAM devices have not yet met the requirements for the mass production of large-scale applications. The fact that these problems remain unsolved is significantly correlated with the insufficient understanding of the underlying switching mechanism. Although some devices have been shown to present area-dependent resistance modulation, RRAM devices based on the formation and rupture of nanoscale conductive filaments (CF) in simple CMOS-compatible binary oxides are the closest to widespread application^9^. In these devices, the formation/rupture behavior of the nanoscale CF is responsible for the observed RS effects, i.e. it controls the SET/RESET transition between the high resistance state (HRS) and low resistance state (LRS). Thus, not only the device performance but also the fluctuation of resistive switching parameters and the related reliability problems are intrinsically related to the microscopic physics of the CF. In these devices, the reaction and movement of metal cations or oxygen anions in the filament region control its geometry and its electrical behavior. As a result, since atomic movements have a deep influence on the structure of the filament, the switching behavior cannot be precisely controlled by the external electric stimuli. In this regard, achieving improved device performance, uniformity, and reliability requires a deeper understanding of the CF dynamics. Considering the stochastic characteristics of the RS process, the Monte Carlo (MC) method has been proved to be effective in analyzing the conductive-insulating transition behavior driven by the electric field^10–12^. Previous atomic-scale MC simulations of RRAM device have focused on specific device structures with particular switching mechanisms, such as electrochemical metallization mechanism (ECM), mainly based on the migration of metal ions^13–15^, or valence change mechanism (VCM) related to oxygen vacancies dynamics in RRAM^16–20^. However, comparing the electric characteristics and statistical results obtained in these different devices, the macroscopic SET behaviors are very similar even if the devices are based on different materials and dominated by different microscopic mechanisms^21–23^. As a consequence, a MC simulator based on a higher level description, as proposed in this work, is necessary and useful.

**Figure 1:**
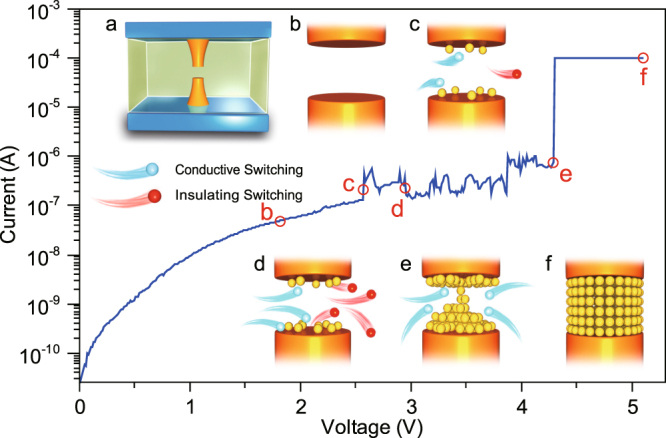
Typical experimental *I* − *V* curve showing abrupt SET switching and the corresponding different stages of CF formation. Inset (**a**) Schematic of a RRAM device with remnant CFs in the insulating layer. Insets (**b**–**f**) Magnified schematics of the gap region in the CF during the SET process, including (**b**) the initial open gap in HRS, the intermediate filament growth with conductive cells gradually (**c**) increasing and (**d**) decreasing and giving rise to current fluctuations, (**e**) the just formed tiny CF with one column of cells connected (quantum wire limit), and (**f**) the strong CF in LRS after the constant *I*_*cc*_ stage, respectively. It is worth noting that the remnant CFs keep comparatively stable during the evolution. This figure also illustrates the geometrical approach of the cell-based model. Our simulator only deals with the dynamic modeling of the CF evolution by considering the gap region (fully insulating in the HRS), which, for convenience, is divided into *n* slices with each slice including *N* cells. The parameter *n* is related to the gap thickness (or gap length) and it is the key parameter in the model.

In a previous work, we developed a cell-based MC simulator for the thermal RESET of CF which is able to explain all the phases of the RESET process. It captures the initial abrupt RESET transition (due to positive feedback between thermal dissipation and conductance reduction), the subsequent progressive phase of conductance reduction, and the final rupture of the CF after reaching the dimension of a single chain of atomic defects^12^. In this work, we focus on the SET transition and develop an analogous simulator for this transition. We depart from the rather well established assumption that the device forming creates a conduction filament which is partially broken during RESET and reformed during SET. We do not consider the possibility that other filaments are formed during SET due to the much reduced SET voltage (with respect to the forming voltage). Thus, we focus on the defect dynamics in the gap region of a single filament after any RESET event. This partially broken CF is responsible for the conduction in the HRS and its reconstruction during the SET process consists in the reconnection of the two stumps remaining after the RESET transition. As shown schematically in Fig. 1, we consider that the CF is formed by cells, i.e. the basic switching units, which can switch between two states: an insulating state (non-defective) and a conductive state (defective). Driven by the externally applied electric field, and under the effect of redox and ion migration, the presence of conductive metal atoms and associated oxygen vacancies can be represented as cells switch from insulating state to conductive one^21, 24–27^, and under ECM, the switching kinetics contains the process of nucleation and filamentary growth^28^. In the CF, the gap region can be regarded as a bottleneck structure, which acts as a potential barrier for the transport of electrons and is correlated to the thickness and width of the gap region. For simplicity, the potential barrier is considered to be parabolic according to the Quantum Point Contact (QPC) model^29, 30^. The switching of cells from conductive to insulating state in the gap region is translated into variations of the electron transport, so that the changes in the *I* − *V* relationship can be related to changes of the gap microstructure. External electrical stimuli are applied such as those during practical operation schemes, and the evolution of the CF gap and the associated electrical properties will be shown to be nicely captured by our model. In this regard, we will explicitly consider operation in the voltage sweeping mode (VSM)^9, 31^, the current sweeping mode (CSM)^9, 31–33^, the constant voltage stress (CVS) mode^34, 35^, and the gate voltage ramp programming (GVR) mode in one-transistor-one-resistor (1T1R) structure^9, 31, 36, 37^. The experimental *I* − *V* and the statistics of SET/RESET transitions and ON/OFF resistances will be shown to be nicely simulated by our simulator thus showing its universal validity. In particular, through statistical analyses, we will show that the simulator is able not only to reflect the randomness and fluctuation of parameters, but also to reveal the device performance hidden in cycle-to-cycle and device-to-device variations.

## Results and Discussion

### Dynamic CF Formation Model

Despite the different filamentary switching mechanisms, including ECM, VCM and thermochemical mechanism (TCM)^3^, the SET current-voltage (*I* − *V*) curves measured under VSM share very similar features. At low voltages, there is a slow current increase with some fluctuations corresponding to the HRS (open gap without conductive defects). An intermediate stage follows where the CF begins to grow (gradually closing gap), an abrupt current increase which is usually identified as the actual SET transition (instantaneous gap close) and, finally, a constant current stage limited by the compliance (filament widening) completely reforms the CF. Fig. 1 shows a typical experimental *I* − *V* curve of a Pt/HfO_2_/Pt device during a SET process and the corresponding schematics at different stages. Driven by the electrical stimuli, chemical reaction and ionic motion occur in the insulating layer, and this is modeled as a growing number of conductive CF cells. These conducting cells gradually fill the gap between the remnant parts of CFs and lead to the increase of the measured current. Meanwhile, negative and positive feedback coexist during the process, and according to ref. 12, the negative feedback mainly results from thermal effect. Due to a local current increase and heat dissipation, the temperature in the filament region increases, and this results in some of the conductive cells switching back to the insulating state. Therefore, the SET process is a dynamic random evolution with the coexistence of conductive and insulating cells that switch back and forth between conductive and insulating states. Finally, as voltage increases, and a critical number of conductive cells accumulate in CF’s gap, it reforms completely and a stable conducting filament is formed, thus yielding to the LRS.

In actual SET experiments, the electric field is generated by the external signal, a time-dependent function which varies according to the different operation schemes. In VSM, for example, a time-dependent voltage *V*(*t*) is applied whereas in CSM it is the evolution of the current *I*(*t*) which is externally imposed. The external electrical stress acts as the driving force to control the inner switching dynamics of the gap cells, which, at the microscopic level, are controlled by chemical reactions and ionic motion. Our MC simulator is built upon two main ingredients, a model for the dynamic cell switching process and electrical transport model that relates the CF microstructure to its electrical response. While the transport model is deterministic (given a shape of the CF, the *I* − *V* characteristics is determined), the switching model must consider stochastic processes which are controlled by the atomic-scale energy potential barriers for reaction and transport. That is the reason behind the use of a Monte Carlo simulation scheme.

#### Cell Switching Model

As-fabricated devices usually require a forming process to induce the switching from the initial (fully insulating) state to the LRS. During forming process, a long conductive channel (>10 nm in most cases) is created through the whole insulating layer, a process which is very similar (if not identical) to a soft-breakdown event (soft, because the current is externally limited). After that, according to the direct observations of Yang *et al*.^38^, the CF tends to be partially ruptured during the RESET process. Thus, in general, the SET process, can be regarded as a dynamic reconstruction of the filament in the gap region opened during RESET, whereas most of the remnant CF remains stable. New filaments are much less likely to form during the SET process because the electric field, current, and temperature are much smaller in the rest of the insulating layer than in the CF gap^29^. Although in Fig. 1, the gap has been arbitrarily located in the middle of the CF, it is worth noting that its position within the CF will depend on the filament shape and on the previous RESET transition^39^. In general, the rupture of the filament during RESET is likely to happen in the narrowest cross-sectional area of the CF due to self-accelerated thermal dissolution^40^.

The microstructure of the CF gap region is illustrated in the insets of Fig. 1. The remnant CF determines the conductive channel, and can be regarded as a small series resistance (about dozens of ohm in most cases) which will not have a strong influence on the SET process because it is much smaller than the resistance of the CF gap. The gap region can be described in the framework of the cell-based percolation model^21, 24^. In the HRS, the gap is filled with insulating material (non-defective metal oxide) and can be modeled as being formed by geometrical cells in the insulating state. Then, driven by external electrical stimuli, reduction reactions and ion motion occur, accompanied by the migration of active metal cations and the subsequent formation of conductive metal atoms (such as Ag, Cu, etc.), or by the movement of oxygen ions which leaves and introduces oxygen vacancies associated to conductive metal atoms with a reduced state. As a result, the insulating cells switch into conductive state. For simplicity, the average “diameter” of a cell is considered to be a constant *a*_0_. For example, in the Pt/HfO_2_/Pt structure, *a*_0_ indicates the distance between neighboring oxygen vacancies in monoclinic HfO_2_^21^. Then the whole 3D space in the gap region can be described by:

1 $${A}_{{\rm{gap}}}=N\cdot {a}_{0}^{2},$$

2 $${t}_{{\rm{gap}}}=n\cdot {a}_{0}.$$

*A*_gap_ and *t*_gap_ are the cross-sectional area and thickness of the gap region, indicated by the number of cells *N* and number of layers *n*, respectively.

As mentioned above, cells are able to switch from insulating state to conductive one under external electric stimuli. For the *i* ^th^ cell, the switching to the conductive state can be described by a SET cumulative probability function (CPF), *F*_*SET*,*i*_. For the mathematical description of this CPF, we depart from the framework of the percolation model developed for the phenomenological description of oxide breakdown^24^. This is possible because, in the end, the SET process is analogous to the breakdown of a tiny insulator (very small area and thickness of the CF gap). In this regard, following the standard phenomenological picture of oxide breakdown, we will consider that defects are generated in the gap cells. The generation of one or more defects in a cell during stress will switch it to the conductive state. The density of defects increases with the stress time and the average number of defects per cell, *n*_DEF_ is consequently a time-dependent variable. Assuming a uniform generation of defects, the CPF can be calculated assuming a Poisson process:^24^

3 $${F}_{{\rm{SET}},i}=1-\exp (-{n}_{{\rm{DEF}}}).$$

In the dielectric breakdown process, when the oxide is stressed under constant voltage, *n*_DEF_ shows a slightly non-linear dependence on time, i.e.,$\,{n}_{{\rm{DEF}}}(t)={(t/{\tau }_{T}(t))}^{\alpha ^{\prime} }$ where *α*′ is a constant and *τ*_*T*_(*t*) is a scale factor which depends on the applied voltage in a strongly non-linear way^41^. For more general stress wave functions, the defect density needs to be expressed as an integral because the applied voltage and local electric filed in the gap change continuously during the SET process:

4 $${n}_{{\rm{DEF}}}(t)={[{\int }_{0}^{t}{\tau }_{T}{(t)}^{-1}dt]}^{\alpha ^{\prime} }.$$

It is not easy to determine which is the law that best describes the dependence of the time-dependent defect generation rate on the applied voltage because the range of voltages that gives rise to a reasonable SET time (not too short and not to long) is very narrow due to the strongly non-linear relation between the breakdown time (the SET time in our case) and the applied voltage. However, in oxides with thickness below approximate 5 nm, a power-law is the best assumption:

5 $${\tau }_{T}(t)={\tau }_{T0}{({V}_{{\rm{gap}}}/{t}_{{\rm{gap}}})}^{-m},$$

where *V*_gap_ is the voltage drop at the gap region, and *m* is the voltage acceleration factor which can be assumed as a constant for simplicity^42^.

As we have already discussed before, the SET process is actually a time-dependent balance between cell switching to conductive and to insulating states, i.e., a dynamic process by which defects are created and annealed. Thus, for the simulation of the SET transition, we also need to include a model for the cell transition from conducting to insulating state. In agreement with experimental results, we have assumed that this process is mainly dominated by thermal dissolution effects^25, 40, 43^. Heat energy generated during the SET process cannot be evacuated instantaneously and hence, there is always an associated heating of the CF region, and this increases the reoxidation and partial dissolution of the CF which somehow compensate the generation of new defects in the CF. According to the analysis of self-accelerated thermal dissolution model, the fastest RESET transition occurs in the bottleneck region which has the smallest cross section^40^. Thus the negative feedback (RESET dissolution) is also expected to occur in the gap region, and this process is governed by RESET probability function *F*_RESET_ given in ref. 12. Therefore, our MC simulation of the growth of the CF during SET operation consists in a dynamic process resulting from the competition between *F*_SET_ and *F*_RESET_. When conductive cells are continuously generated and a column of cells switch into conductive state, as illustrated in Fig. 1e, the gap disappears and the two electrodes are finally connected by a conductive channel. The resistance of the whole device would be dramatically reduced which can be observed as a current jump in the SET point. Then if the electric stress is still applied, all the cells in the region will switch into conductive state (Fig. 1f) with the diameter of CF increasing and a strong conductive channel will be fully formed.

#### Electrical Transport Model

During the SET process, the gap region, composed by insulating cells, acts as a potential barrier that limits the flow of electrons, which is controlled by quantum-mechanical tunneling. Therefore, Ohm’s law (which is often valid in the LRS) is not applicable, and the current becomes a strongly nonlinear function of the applied voltage. According to the QPC model, the conduction properties of the CF are determined by electron transport through a very narrow constriction, and it can be regarded as a quasi-one dimensional system^29, 30^. If the CF has no gap, conduction is essentially linear and might show conductance quantization effects if the CF is narrow enough^44, 45^. If there is a gap, transport is by tunneling with an electron transmission probability *T*(*E*) through a potential barrier with height *Φ* and thickness *t*_b_^29^. According to ref. 29, the transmission can be expressed as:

6 $$T(E)={\{1+\exp [-\alpha (E-\Phi )]\}}^{-1},$$

where $\alpha ={t}_{{\rm{b}}}{\pi }^{2}{h}^{-1}\sqrt{2{m}^{\ast }/\Phi }$ is a constant related to the potential barrier curvature and m* is the effective electron mass of electrons in the conducting channel. Under application of an external voltage *V*, and assuming that the non-equilibrium distribution functions of electrons in the metal reservoirs can be approximated by the Fermi-Dirac distribution *f*(*E*) with Fermi levels shifted by the applied potential (Landauer-Datta approach to ballistic transport), the current can be calculated as the integral over electron energy *E*:

7 $$I=\frac{2e}{h}{N}_{c}{\int }_{-\infty }^{\infty }T(E)\{f[E-e\beta V]-f[E+e(1-\beta )V]\}dE.$$

where *e* and *h* are the electron charge and Planck constant, respectively, and a fraction of the external voltage *βV* is assumed to drop at the cathode interface and a fraction (1 − *β*)*V* at the anode one. Considering the equivalent circuit diagram^28^, *V*_gap_ can be related to the applied voltage *V* with the expression of *V* = *V*_gap_ + *IR*_*s*_, where *R*_*s*_ is the series resistance originated from remnant CF, cables and contacts of the experimental setup, etc. *N*_*c*_ is the number of unidimensional conduction modes or, in other terms, the number of 1-D CFs, each one consisting of a single chain of conducting metal atoms (or oxygen vacancies) and having a conductance of the order of the quantum conductance (*G*_0_ = 2*e* ^2^/*h*). Integrating this equation under the assumption of *T* = 0 K, for simplicity, the current is related to voltage as:^29^

8 $$I=\frac{2e}{h}{N}_{c}\{eV+\frac{1}{\alpha }\,\mathrm{ln}[\frac{1+\exp \{\alpha [\Phi -e\beta V]\}}{1+\exp \{\alpha [\Phi +e(1-\beta )V]\}}]\},$$

Therefore, the current flow *I* = *I*(*V*, *Φ*, *α*) is determined by the external voltage *V* and the internal barrier parameters (height *Φ* and thickness *t*_b_), which are related to the gap constriction geometry^29^. The nanoscale gap between the remnant CF stumps can be described as a 1D parabolic potential barrier^46^ with barrier height equal to the difference between the bottom of the first energy subband and the Fermi level at the cathode, and thickness defined as the barrier width at *E* = *E*_*F*_^46^. The assumption of a parabolic barrier was made in previous work because it provides and analytical expression of the transmission coefficient as given by Eq. 6. However, this assumption can be further justified and optimized by considering the combined effects of applied electric field and image force barrier lowering^47^, and these influences on barrier parameters are discussed in the Part 1 of Supplementary Information with Fig. S1.

#### Monte Carlo Simulator description

**Figure 2:**
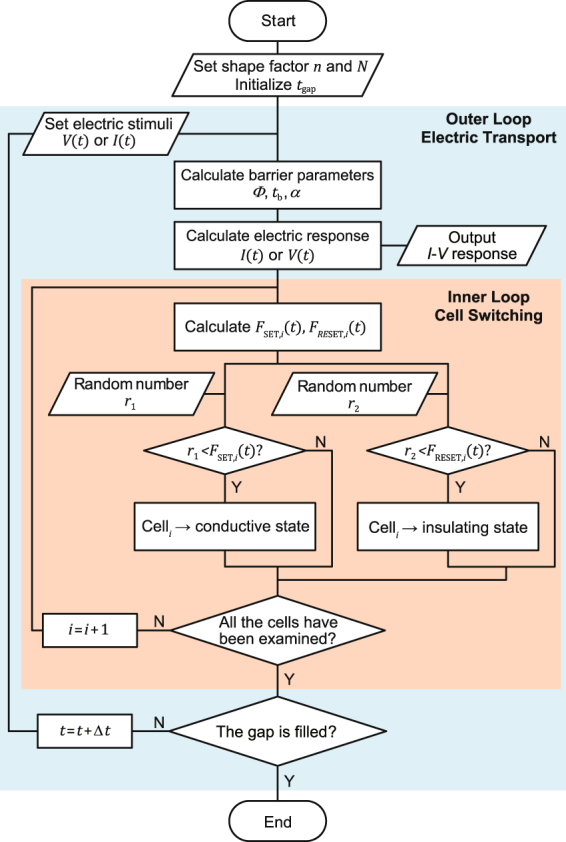
Flow chart of the Monte Carlo simulator for SET process.

Based on the SET/RESET probability distribution functions and the QPC transport model, a dynamic Monte Carlo simulator has been developed for the SET switching process (see the flow chart in Fig. 2). The SET process is simulated with two nested loops: the electrical stimuli and the time evolution are implemented in the outer loop; whereas in the inner loop, the switching process between insulating and conductive cell states is determined for each cell in turn, progressively giving rise to the evolution of the CF geometry. Since in actual experiments, the CF parameters may vary within a certain range between different switching cycles, the initial value of *n* is randomly set at the first stage. According to the statistical results of the soft breakdown experiment of Suñé and Miranda^48^, the parameters of the potential barrier slightly fluctuate within narrow ranges, so that the barrier height *Φ* and curvature *α* are set to follow Gaussian distributions with standard deviation of 5% and 10%, respectively. In the inner loop, since the conductive switching occurs with the SET probability, conductive switching in each cell is realized when *F*_SET,*i*_ is larger than a random number *r*_1_. On the other hand, based on the RESET Monte Carlo simulator previously developed in ref. 12, the insulating switching of a cell is determined by comparing *F*_RESET,*i*_ with a second independent random number *r*_2_. At the final stage, when the conductive channel is fully established (all the cells in the gap having switched to their conductive state), the simulation will be terminated.

### Simulation of the SET transition under different electrical stress conditions

External electrical stimuli are required to cause the evolution of the nanoscale CF. Different operation schemes determine how the CF growing process takes place, resulting in various evolutions of the *I* − *V* characteristics. Moreover, electrical operation and measurement is one of the most important parts in the performance evaluation of RRAM devices^9^, and the subsequent statistical analyses reveal the random disturbances inherent to the SET/RESET process. In this section, the Monte Carlo simulator introduced above will be used to simulate performance under different operation schemes, thus demonstrating that it can handle any kind of stress conditions. After this, statistical analyses of the simulation results are illustrated and compared with experimental results, in order to test the simulator’s validity and universality.

#### Voltage Sweep Mode Operation

**Figure 3:**
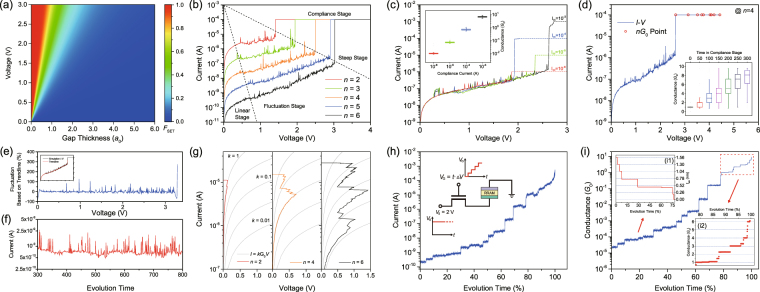
Simulation results obtained from various operation schemes. (**a**) Heat map of the SET probability for different gap thickness *t*_gap_ and gap voltage *V*. Plots of different *I* − *V* characteristics under (**b**–**d**) VSM, (**g**) CSM and (**h**,**i**) GVR operation schemes. (**c**) and (**d**) *I* − *V* characteristics corresponding to different values of *I*_*cc*_ and different stress times in the compliances state (i.e. at *I* = *I*_*cc*_), respectively. The insets of (**c,d**) are statistical results of conductance. (**e**) Fluctuations of current under VSM. The inset exhibits the simulation result of *I* − *V *characteristics (black line) and its trendline (red line). (**f**) Simulated tested *I* − *t* curve in Cu/HfO_2_/M structure. In the simulation, a constant voltage stress of 1 mV is used, close to that used in the experiment. Inset of (**h**) The schematic of the circuit diagram in the GVR mode. (**i**) The plot of conductance jump corresponding to (h). Inset (**i1**) The evolution of the gap thickness when *G* < *G*_0_ (the CF gap is not yet completely closed). Inset (**i2**) The magnified plot of conductance when *G* > *G*_0_, and the conductance changes show discrete quantum effects.

VSM is one of the most frequently used methods for the electrical test of RRAM. In this operation scheme, an ever-increasing voltage is applied with a constant rate *V*(*t*) = *R*_*V*_ · *t* and the *I* − *V* characteristic evolves in four distinctive stages (Figs. 1 and 3b): the current linearly rises up with comparatively stable resistance at first (Linear Stage); then it starts to fluctuate and its amplitude gradually increases (Fluctuation Stage); finally, the current dramatically jumps up by several orders of magnitudes (Steep Stage) and the CF continues to grow under fixed compliance current (*I*_*cc*_) until a strong CF is formed and the device has been fully switched into the LRS (Compliance Stage). The schematics of the *n* × *N* cells’ evolution describing the whole initial CF gap are shown in Fig. S15 of the Supplementary Information, to illustrate the relation between geometry and conduction properties along the whole SET process. The simulation results in Fig. 3b nicely reproduce the experimental results of Long *et al*. (see Fig. S2 in the Supplementary Information)^21^, and the whole process can be understood in the framework of our model as explained below. In the Linear Regime, the SET probability (*F*_SET_) is relatively low, and the gap region remains stable (Figs. 1b and S15a in the Supplementary Information). In the Fluctuation Regime, as time evolves and the probability rises to a larger degree, conductive switching in some of the cells occurs (Figs. 1c and S15b, c in the Supplementary Information). The dimension of gap region shrinks and leads to a progressive decrease of the barrier parameters, accompanied with a current increase. On the other hand, following the accumulation of dissipated energy in the form of heat, high temperature induces larger RESET probability (*F*_RESET_) and insulating switching is activated (Figs. 1d and S15d in the Supplementary Information). When some of the conductive cells come back to the insulating state, the current drops down, preventing the gap region from further filled by conducting cells. Therefore, the current fluctuations are mainly induced by the competition of positive and negative feedbacks between SET and RESET transitions of individual cells. Then in the Steep Regime, when the voltage increases above a certain soft threshold, *F*_SET_ is dramatically enhanced, as illustrated in the heat map of Fig. 3a. Then if some of cells switch into conductive state, the large *F*_SET_ will lead to a continuous switching behavior. Therefore, a self-accelerated process occurs which leads to the observed “abrupt” SET (Figs. 1e and S15e in the Supplementary Information). Finally in the Compliance Stage, conductive cells in the CF continuously generate, until a strong and stable CF is formed (Figs. 1f and S15f in the Supplementary Information).

Moreover, our simulations with various initial values of *t*_gap_ (realized by setting different initial value of *n*) allow to establish the relationship between the initial *t*_gap_ (directly related to the initial value of *R*_OFF_) and final SET voltage (*V*_SET_). Larger gap length usually gives rise to a higher *V*_SET_, which is also supported by experimental results^21^. This can be explained by the fact that under a certain applied voltage, the electric field defined as *V*/*t*_gap_ is weaker for larger gap thickness, and it is harder to induce the SET process due to the strongly non-linear relation between field and defect generation (see Eqs. 3–5). The SET point is defined as the point at which all the cells in at least one column have switched to the conductive state. Hence, the cumulative probability distribution of the SET transition is given by:

9 $${P}_{{\rm{SET}}}=1-{(1-{{F}_{{\rm{SET}}}}^{n})}^{N}.$$

Here we assume all the cells share the same SET probability (Actually, each cell has its own SET probability *F*_SET,*i*_, and this assumption is just for demonstrating the influence of *F*_SET_ and *n* on *P*_SET_). Referring to the definition of *F*_SET_, larger initial *t*_gap_ often correlates with smaller *F*_SET_. And with more insulating cells in one column, it generally requires higher electric stimuli and longer time to realize the conductive switching of all these cells.

In the VSM scheme, the self-accelerated positive feedback process leads to the abrupt SET transition and may result in hard breakdown, which will damage the device permanently. In order to ensure a stable performance, current compliance *I*_*cc*_ is usually necessary (*I*_*cc*_ is set at 10^−4^ A in our simulation if not otherwise specified). The compliance current stage favors the switching of cells to the conductive state. The *I* − *V* characteristics during VSM stress with varying values of *I*_*cc*_ (from 10^−6^ to 10^−3^ A) are shown in Fig. 3c, and the comparison with experimental results is shown in Fig. S3 in the Supplementary Information. Obviously, the magnitude of the compliance limit will influence the shape of CF and result in different final values of CF conductance. The conductance values at the beginning of the Compliance Stage are extracted, and the statistical results of 200 simulated cycles are shown in the inset of Fig. 3c. This figure demonstrates a linear relation between conductance of CF and *I*_*cc*_, and this means that different resistance states can be achieved by this method. On the other hand, by controlling compliance time in the Compliance Stage, resistance states can also be tuned (Fig. 3d). However, this is not a good method to achieve well-defined discrete conductance states because the statistical distributions of final conductance overlap each other (inset of Fig. 3d). In any case, since few studies have considered the influence of compliance time in the compliance state as a method to control CF conductance, more efforts on this subject might end up with an effective method for RRAM devices to realize multi-state storage.

The MC technique is very useful to study noise. In this work, we show that our simulator is capable to reproduce some fluctuations of the *I* − *V* observed during VSM operation. This noise is highlighted by subtracting the average *I* − *V* value from the simulated I-V curve. Eventually these fluctuations might look as Random Telegraph Noise (RTN), as showing in Fig. 3e. Moreover, similar characteristic can be found under CVS mode (Fig. 3f), and the comparison with experimental results is shown in Fig. S4 in the Supplementary Information. With constant stress, two-level or multilevel fluctuations are clearly observed as time evolution. The origin of these fluctuations, in our model, is the interplay of SET/RESET probabilities.

#### Current Sweep Mode Operation

In the traditional VSM scheme, performance is hard to control because of the voltage division in series resistance and device resistance and also because of the parasitic device capacitance. Moreover, the external measuring equipment may cause overshoot effects. CSM was adopted by Naueheim *et al*.^32^ using a current source with constant ramp: $I(t)={R}_{I}\cdot t$, so the current compliance would no longer be necessary. The simulated *I* − *V* characteristics for different initial values of *n* are shown in Fig. 3g. Contrary to what happens during VSM stress conditions, in the CSM, as the time evolves, several voltage drop points can be found, which is also consistent with previously reported experiments^31^. Based on our model, when some of the cells switch into conductive ones, the potential barrier becomes more transparent to electron transport so that less voltage is required to sustain the same current level. It explains why the voltage drops down. Moreover, considering the effect of the series resistance, the voltage drop in the device resistance would be even further reduced^49^. It is worth mentioning that, since the power consumption (assumed to be *I* · *V*) decreases when the applied voltage drops to keep the current constant, less heat will be dissipated and, as a consequence, *F*_RESET_ also decreases. In summary, after each voltage drop, which corresponds to a partial RESET, a negative feedback mechanism reduces the probabilities of cell switching (both SET and RESET) and the CF will not be significantly changed until the current reaches a high enough value (The current increases because a current ramp is applied.). Since the SET process is self-controlled under CSM conditions, more uniform resistive switching parameters (SET voltage, SET current, and final resistance) can be found^33^. This is the main advantages of using CSM conditions for RRAM characterization and operation.

#### Gate Voltage Ramp Programming Operation

The 1T1R structure is adopted in RRAM arrays to effectively suppress the current overshoot related to parasitic capacitance, to set up a reliable current compliance, and to eliminate the sneak-path problem during reading. In the conventional programming scheme, a constant voltage is applied to the gate whereas the varying voltage applied to the source is ramped. These stress conditions are effective in limiting the current due to transistor’s saturation. However, in this stress mode a current jump still takes place, similar to that happens under VSM conditions. On the contrary, in the recently proposed GVR scheme, current jump up to several orders of magnitude will not occur, as observed both in the results of our simulation (Figs. 3h and i) and in the previous experiments of Lv *et al*. (see Figs. S5 and S6 in the Supplementary Information)^37^. In the GVR mode, when a conductive switching occurs, RRAM’s resistance drops down. Then due to transistor’s self-compliance characteristic, decrease of voltage division on the device temporarily suspends the increase of SET probability, similar to what happens in CSM. Therefore, the time evolution will be slowed down and each switching event is observed in an “atomic jump”. The schematics of the *n* × *N* cells’ evolution during the whole process are also shown in detail in Fig. S16 in the Supplementary Information. The evolution of the gap thickness is shown in the inset (i1) of Fig. 3i, and quantized characteristics can be found, i.e., the gap thickness presents step-like changes in the values of the integral multiples with a basic length ~0.26 nm, which is close to the cell size setup in our simulation. This result is in agreement with the experimental report in ref. 37 (see Fig. S14 in the Supplementary Information), where the basic length represents the distance between two adjacent interstitial sites in the HfO_2_ lattice. On the other hand, conductance quantization can be observed at the final stage of the SET switching process, as shown in the inset (i2) of Fig. 3i, which is very similar to the experimental observations reported in ref. 37 (see Fig. S6 in the Supplementary Information). When all the cells in one column switch into the conductive state, the barrier in this column collapses due to the gap’s elimination. Based on the first-principle calculations of transport properties^50^, the conductance of conductive channels is in the order of *G*_0_, a result that can also be derived from the approximation of Eq. 8. Then the conductance jumps can be explained by the continuous formation of multiple conductive channels.

### Simulation of the SET statistics under different operation modes

#### Voltage Sweeping Mode Operation

**Figure 4:**
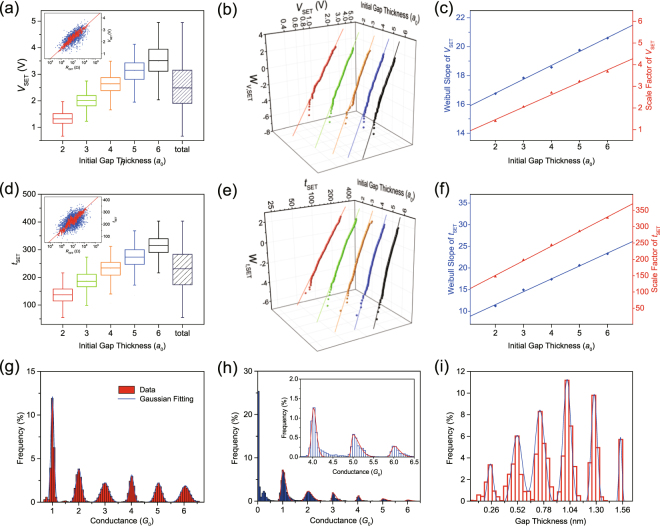
Statistical results of Monte Carlo simulation under VSM, CSV and GVR. (**a**) Box plot of *V*_SET_ extracted from 3000 cycles for different initial values of *n*. The distribution of all the points is shown in the “total” column. Inset of (**a**) The scatter plot showing the relationship between *R*_OFF_ and *V*_SET_ (blue dots) and moving geometric mean (red dots), by calculating the geomean of 20 adjacent voltage values. (**b**) Weibull distributions of *V*_SET_ in 3000 cycles for different *n*. (**c**) Weibull slope and scale factor extracted from (**b**). Both parameters are linearly dependent on *n*. (**d**–**f**) The distribution of *t*_SET_ under CSV. (**g**,**h**) Histogram of conductance distribution (when *G* > *G*_0_) in 100 continuous cycles under VSM and GVR, showing Gaussian distribution and skew normal distribution, respectively. Inset of (**h**) The magnified histogram when *G* > 3.5*G*_0_. (i) The distribution of gap thickness (when *G* < *G*_0_).

The Weibull distribution is widely used for statistical analyses in the area of reliability engineering and survival analysis, and it has also been proved to be adequate for the modeling and analysis of the statistics of switching voltage^21^ and switching time^26^ in the SET transition of RRAM. Considering *y* as the independent variable, the Weibull distribution function can be expressed as:

10 $$W(y)=\,\mathrm{ln}\{-\mathrm{ln}[1-P(y)]\}=\beta ^{\prime} \mathrm{ln}(y/{y}_{63 \% }),$$

where *β*′ is the Weibull slope, a measure of the distribution’s dispersion, and *y*_63%_ is the scale factor, which is defined by $W({y}_{63 \% })=0$ and this corresponds to $P({y}_{63 \% })\approx 0.63$. From the experimental results of Long *et al*.^21^ the distribution of *V*_SET_ under VSM was proved to follow the Weibull distribution, and a data screening method was adopted to suppress the interference caused by variation of *R*_OFF_ (resistance under HRS), as it is also found in our simulated results shown in the box statistical plot shown in Fig. 4a. Here, simulations with different initial gap thicknesses were performed and, as shown in Fig. 4a, for each initial value of *n*, the distribution of *V*_SET_ is much narrower than that corresponding to the global data. Furthermore, the average *V*_SET_ value increases with initial *n*, in agreement with the experimental results (see Fig. S7 in the Supplementary Information)^21^. Fig. 4b shows the simulation results of *V*_SET_ distributions corresponding to 3000 cycles in the Weibull plot, showing that also the results of our simulations satisfactorily fit the Weibull distribution^21^ (see Fig. S8 in the Supplementary Information).

In fact, departing from Eq. 9 for the SET probability distribution *P*_SET_ and considering the limit *F*_SET_ << 1 for simplicity, and coupling Eqs. 3–5, an analytical approximation for the SET voltage distribution can be obtained:

11 $$W({V}_{{\rm{SET}}})\approx {\beta }_{{V}_{{\rm{SET}}}}^{^{\prime} }\,\mathrm{ln}({V}_{{\rm{SET}}}/{V}_{{\rm{SET}}63 \% }).$$

It can be straightforwardly shown that the Weibull slope ${\beta }_{{V}_{{\rm{SET}}}}^{^{\prime} }$ and the scale factor *V*_SET63%_ are linearly dependent on *n*, i.e. the initial gap thickness. Fig. 4c shows the corresponding simulation results (without the approximations implicit in Eq. 11), that show similar trends with this simple analytical picture and excellent consistency with the experimental results^21^ (see Fig. S9 in the Supplementary Information). It’s worth noting that the conductance distribution is shown in Fig. 4g, and the values mainly concentrate on multiple orders of *G*_0_, following Gaussian distributions around these values, which is similar to the previous experimental results^44^.

#### Constant Voltage Stress Operation

CVS mode can be regarded as an effective method to test the switching time, read-disturb time and retention behavior. With the purpose of timely monitoring the resistance state, a width-adjusting pulse operation (WAPO) method^26^ was recently implemented by Zhang *et al*. to accelerate the experimental determination of the distribution of SET switching time (*t*_SET_)^26^. In this method, rectangular pulses with constant height and increasing width are generated, and small read pulses are applied between programming pulse intervals to acquire the resistance value and examine whether the SET operation succeeds. This experimental method allows to accelerate the SET process and the extraction of the *t*_SET_ versus *V*_SET_ relationship. However, in our simulation scheme, we can directly simulate simple constant-voltage stresses with “real-time” monitoring of the resistance during the switching process. Figs. 4d and S10 in the Supplementary Information show the box statistical plot of the simulated *t*_SET_ data, whose characteristics are rather similar to those reported in Fig. 4a for VSM operation, but now the switching variable is *t*_SET_. Combining Eqs. 3–5 and 9, the *t*_SET_ distribution can be derived in the same approximate conditions as Eq. 11. The result is also a Weibull distribution of *t*_SET_ with Weibull slope ${\beta }_{{t}_{{\rm{SET}}}}^{^{\prime} }\,$and scale factor *t*_SET63%_ which again are both proportional to *n*. Fig. 4e illustrates the Weibull distributions of *t*_SET_ for a sample population of 3000 cycles with different initial *n*. Once again, the simulation results are in excellent agreement with the experimental results (see Fig. S11 in the Supplementary Information)^26^. The simulated Weibull slope and scale factor also exhibit a linear dependence on *n*, as shown in Fig. 4f, and in excellent agreement with experiment (see Fig. S12 in the Supplementary Information)^26^.

According to the previous studies, the resistance in HRS can be related to the gap thickness *t*_gap_ as^21^

12 $${R}_{{\rm{OFF}}}=\frac{1}{{G}_{0}N}\exp ({t}_{{\rm{gap}}}/{t}_{0}),$$

where ${t}_{0}=\frac{h}{\pi }\sqrt{\frac{2}{{m}_{0}{\Phi }_{0}}}$. The gap thickness in turn is proportional to *n* so it is straightforward that *n* and *R*_OFF_ are logarithmically related, $n\propto \,\mathrm{ln}({R}_{{\rm{OFF}}})$. Thus, it is evident that our statistical simulation results reported as a function of *n* in Fig. 4a–f are equivalent to the experimental results reported as a function of *R*_OFF_. This equivalence is also demonstrated in the semi-log plots shown in the inset of Figs. 4a and d (Figs. S6 and S9 in the Supplementary Information show the comparison of simulations with experimental results.), where *V*_SET_ and *t*_SET_ are found to logarithmically increase with *R*_OFF_. This type of results have been experimentally reported in a variety of different RRAM devices which are based on various switching mechanisms. In particular, analogous results have been reported in ECM device with Cu/Ta_2_O_5_/Ru^23^ or Cu/HfO_2_/Pt^27^ structure, in bipolar VCM devices with TiN/HfO_x_/Pt^22^ or Ti/ZrO_2_/Pt^26^ structure and also in unipolar VCM devices with a Pt/HfO_2_/Pt structure^21^. This example reveals the universality of the results of our model, which being based on a somehow compact (behavioral) approach to the microscopic mechanisms, allows more general predictions to be made than atomic-scale MC simulators which are more device specific.

#### Gate Voltage Ramp Programming Operation

As discussed in the last section, quantum conductance effects can be observed in the GVR operation scheme. In order to observe the statistical results of conductance distribution, values of conductance corresponding to 100 cycles are extracted into a global histogram (Fig. 4h). The values mainly concentrate on multiple orders of *G*_0_, following skew normal distributions around these values. Moreover, higher statistical frequencies are found in the lower conductance region. This can be attributed to the fact that the system remains shorter time in higher conductance states because switching is faster in these states. The simulation results are consistent with the recent experimental results of Lv *et al*. (see Fig. S13 in the Supplementary Information)^37^. These results also prove that the SET switching process is a self-accelerated process that can be effectively slowed down (multiple steps rather than abrupt jump) by the GVR scheme.

## Conclusion

In this study, a compact Monte Carlo simulator is developed for the SET process in RRAM devices, based on a cell description of the geometry of the gap of the conducting filament. This type of MC simulator somehow weakens the microscopic detail of the atomic-scale physics to gain universality, i.e., to be applicable to different types of devices based on different microscopic mechanisms. In order to test the validity and universality of our simulator, simulations based on various practical operation schemes in different types of RRAM devices have been reported. The dynamic evolution of the *I* − *V* characteristics during the SET process has been shown to be in excellent consistency with the experimental results. Macroscopic electrical time-dependent response under the different operation modes reflects the changes in the dynamic evolution of the CF in its gap region. Moreover, statistical analyses also reflect the underlying properties of resistive switching parameters. The initial resistance value has a strong relationship with *V*_SET_ and *t*_SET_ at the final stage. Their statistical distributions are shown to be nicely captured by the Weibull distribution model and also in complete agreement with experiments. In the simulation of 1T1R structure under GVR mode, quantized discreteness of the CF’s conductance distribution is revealed, also in perfect agreement with recent experiments. In summary, our cell-based MC simulation procedure has been demonstrated to nicely capture the dynamic evolution of the SET switching process, in the same way that our previous work demonstrated for the RESET process. Coupling of our SET/RESET cell-based simulators might be an excellent tool to guide other researchers in the understanding of the dynamics of the switching behavior in RRAM devices to a deeper extent. Moreover, it can be a powerful design tool to define optimized methods for SET and RESET processes in practical applications.

## Electronic supplementary material

Supplementary Information
