# Supplementary material for: Investigation on the Conductive Filament Growth Dynamics in Resistive Switching Memory via a Universal Monte Carlo Simulator
Source: Sci Rep. 2017 Sep 11;7:11204. doi: 10.1038/s41598-017-11165-5 (PMC5593871; doi:10.1038/s41598-017-11165-5)
Supplement: Supplementary file 1 — Supplementary Information [file 41598_2017_11165_MOESM1_ESM.pdf]

Supplementary Information

Investigation on the Conductive Filament Growth Dynamics in Resistive Switching Memory via a Universal Monte Carlo Simulator

Yu Li[1,2,4,5], Meiyun Zhang[1,4,5], Shibing Long[1,4,5,*], Jiao Teng[2], Qi Liu[1,4,5], Hangbing Lv[1,4,5], Enrique Miranda[3], Jordi Suñé[3] and Ming Liu[1,4,5,]**

[1]Key Laboratory of Microelectronics Devices & Integrated Technology, Institute of Microelectronics of Chinese Academy of Sciences, Beijing, 100029, China

[2]University of Science and Technology Beijing, Beijing, 100083, China

[3]Departament d'Enginyeria Electrònica, Universitat Autònoma de Barcelona, Bellaterra 08193, Spain

[4]University of Chinese Academy of Sciences, Beijing, 100049, China

[5]Jiangsu National Synergetic Innovation Center for Advanced Materials (SICAM), Nanjing 210023, China

Correspondence and requests for materials should be addressed to S.L. (email: longshibing@ime.ac.cn) or M.L. (email: liuming@ime.ac.cn)

1. Barrier Parameters Modulation with Image Force Barrier Lowering Effect

The combined effects of applied electric field and image force barrier lowering will induce the change of barrier's shape, resulting in the change of barrier height Φ and barrier thickness t_{b} to some extent. Therefore, different from previous studies, during the switching process, the barrier parameters are no longer constant but are tuned by external electric field and internal microstructure of gap region.

We can regard the gap region within the two remnant stumps of the CF as an ultra-thin insulating film sandwiched between two local "electrodes". The applied electric field and the image force further modify the shape of the barrier. The analytic solution of the image force potential energy $U(x)$ in the position x of the gap region $(0 < x < t_{\mathrm{gap}})$ is given by[s1]

$$U(x) = \left(-\frac{e^2}{4\pi\varepsilon}\right)\left\{\frac{1}{2x} + \sum_{l=1}^{\infty}\left[\frac{l\cdot t_{\mathrm{gap}}}{(l\cdot t_{\mathrm{gap}})^2 - x^2} - \frac{1}{l\cdot t_{\mathrm{gap}}}\right]\right\}, \tag{1}$$

where e is the electron charge, ε is the permittivity of the gap material and t_{gap} is the thickness of the gap region. When an external voltage V is applied, the barrier will be further lowered under the influence of electric field. Considering these two effects, the generalized formula for the effective barrier height is

$$\Phi(x) = \Phi_0 - \frac{eVx}{t_{\mathrm{gap}}} + U(x). \tag{2}$$

After that, the barrier parameters can be conveniently extracted: the actual thickness t_{b} can be simply represented as the result of subtraction between two ends of barrier x_1 and x_2, which are two real roots of $\Phi(x) = 0$; the height of the barrier can be represented as the maximum of $\Phi(x)$, for simplicity. Figure S1 exhibits the barrier under the influences of image potential and applied voltage. According to the results of calculation, the shape of barrier is changed by variations of the inner gap thickness (Figure S1e) and external applied voltage (Figure S1f), accompanied with the corners of rectangular rounded off and both height and thickness reduced.

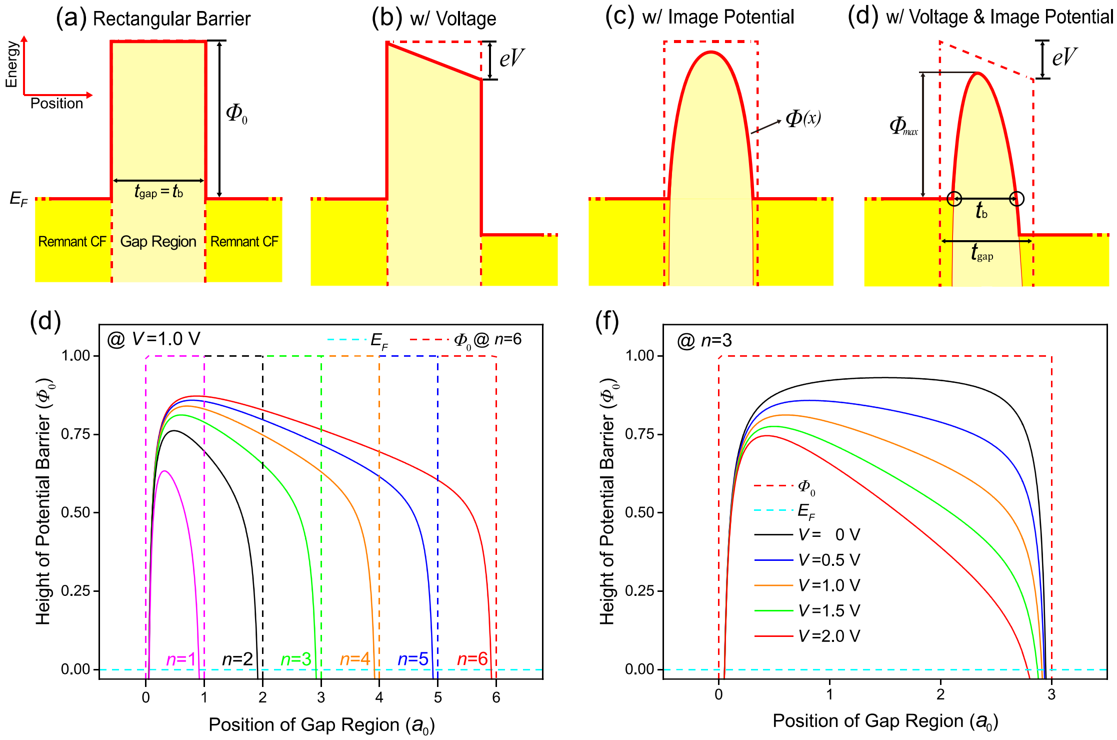

Figure S1 | Energy diagram of the potential barrier for different values of gap thickness and/or the amplitude of the applied voltage. (a) Rectangular potential barrier in the conducting filament gap region. **(b)** Potential barrier under applied voltage. **(c)** Potential barrier under the influence of the image force. The corners are rounded off and both the barrier height and thickness are reduced. **(d)** Potential barrier under the superposed effects of the external voltage and the image force. **(e)** Different barriers corresponding to a gap with different number of cells n (voltage is fixed at 1 V). **(f)** Different barriers changing with the applied voltage V (n=3). Comparing with the original rectangular barrier (dashed lines), the decrease of barrier thickness is not significant, while the height dramatically drops down. Roughly estimated, when the gap thickness reduces to the width of only one cell, or the voltage is high enough (~2 V), the effective barrier height will be less than 75% of Φ_0.

2. Comparison of MC Simulation Results and Previous Experimental Results

2.1. SET I-V curves under different electrical stress conditions

2.1.1. Voltage Sweep Mode Operation

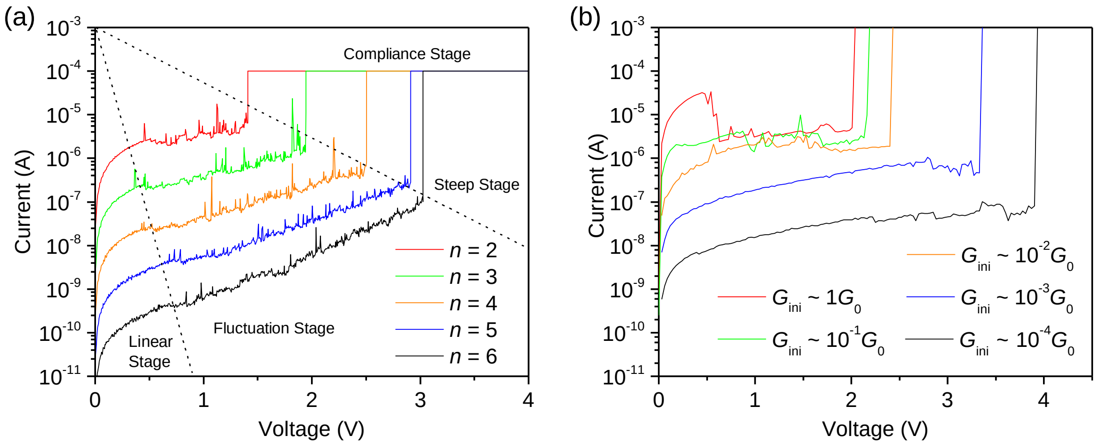

Figure S2 | Comparison of $I-V$ responses under VSM in **(a)** MC simulation (Figure 3b in the main text) and **(b)** previous experiment.[s2]

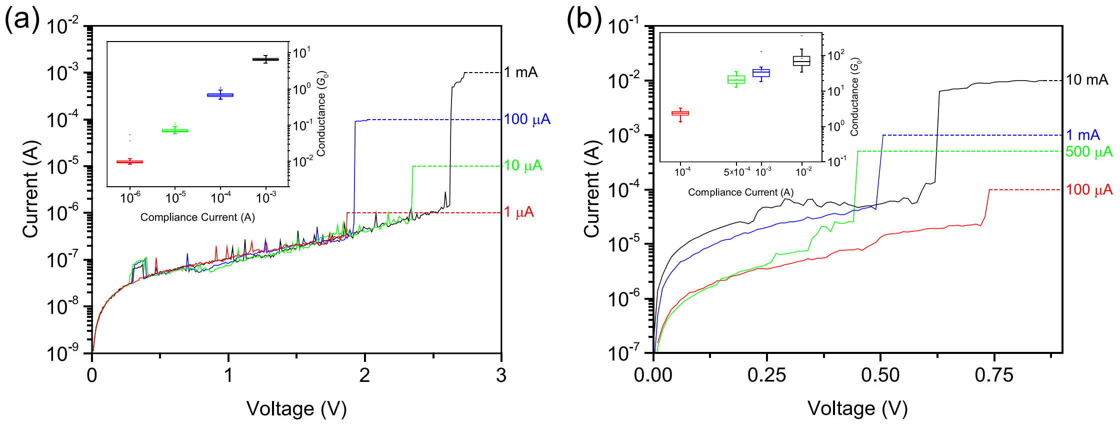

Figure S3 | Comparison of $I-V$ characteristics under VSM with different values of I_{cc} in **(a)** MC simulation (Figure 3c in the main text) and **(b)** previous experiment.[s3]

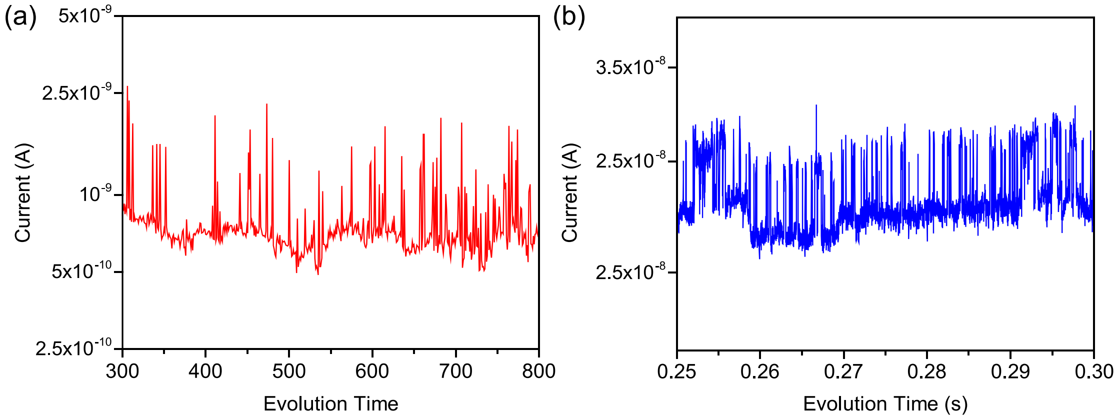

Figure S4 | (a) Simulated and **(b)** experimentally tested $I-t$ characteristic in Cu/HfO_2/M structure. In the simulation (Figure 3f in the main text), a constant voltage stress of 1 mV is used, close to that used in the experiment.[s4]

2.1.2. Gate Voltage Ramp Programming Operation

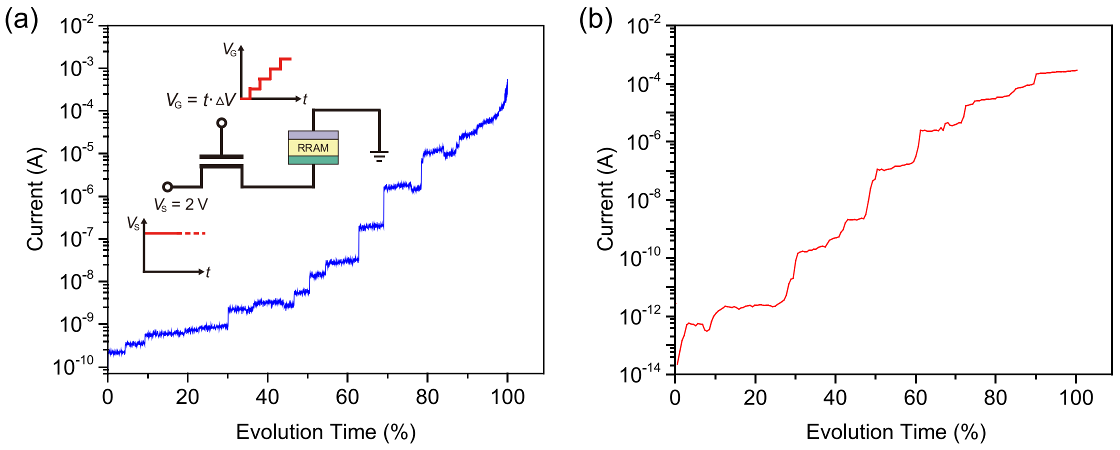

Figure S5 | Comparison of $I - V$ responses under GVR in **(a)** MC simulation (Figure 3h in the main text) and **(b)** previous experiment.[s5]

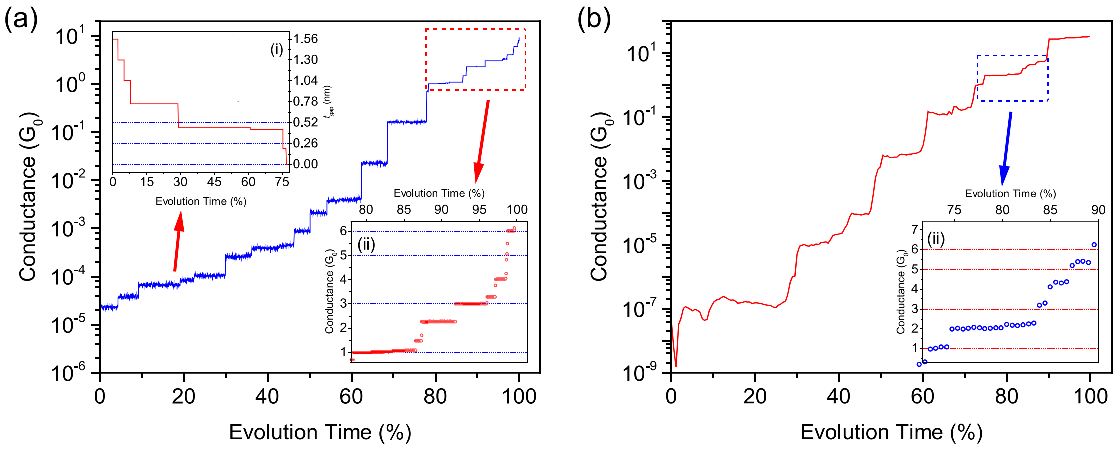

Figure S6 | Comparison of conductance/resistance as well as conductance variation at the final stage under GVR in **(a)** MC simulation (Figure 3i in the main text)and **(b)** previous experiment.[s6]

2.2. SET statistics under different operation modes

2.2.1. Voltage Sweeping Mode Operation

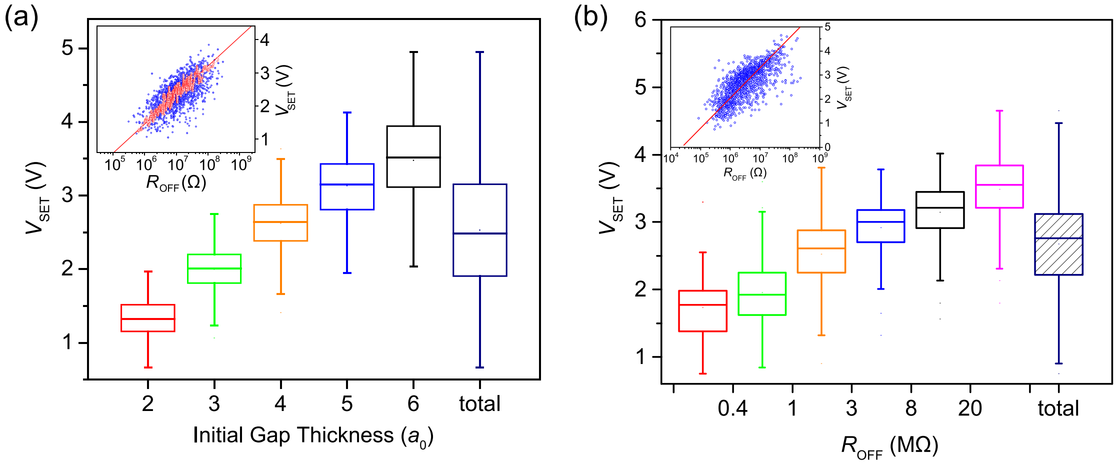

Figure S7 | Comparison of statistical results of $V_{\rm SET}$ distribution under VSM in **(a)** MC simulation (Figure 4a in the main text) and **(b)** previous experiment.[s2]

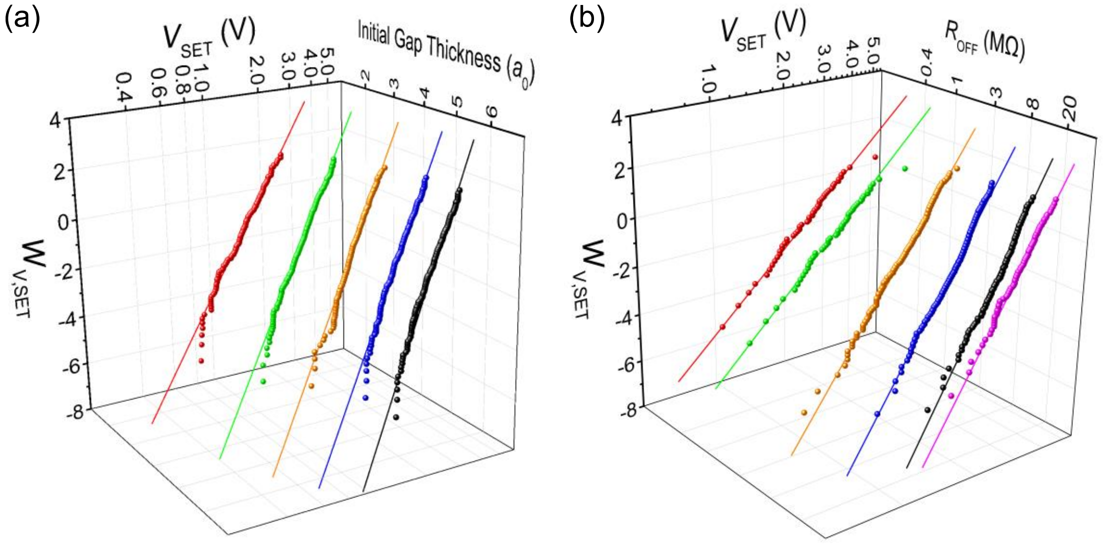

Figure S8 | Comparison of Weibull distribution of V_{SET} under VSM in **(a)** MC simulation (Figure 4b in the main text) and **(b)** previous experiment.[s2] Experimental results are plotted versus $\log(R_{\mathrm{OFF}})$ which, as discussed in the text, is proportional to n.

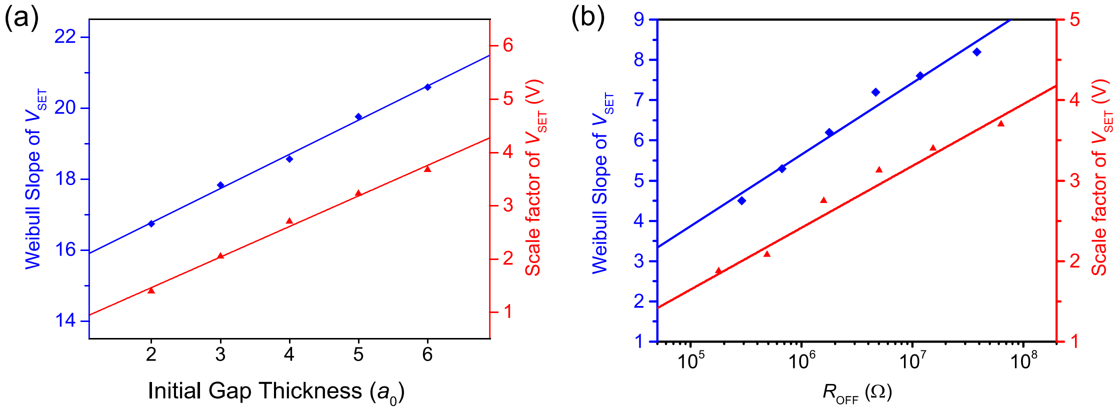

Figure S9 | Comparison of Weibull slope and scale factor extracted from V_{SET} distribution under VSM in **(a)** MC simulation (Figure 4c in the main text) and **(b)** previous experiment.[s2]

2.2.2. Constant Voltage Stress Operation

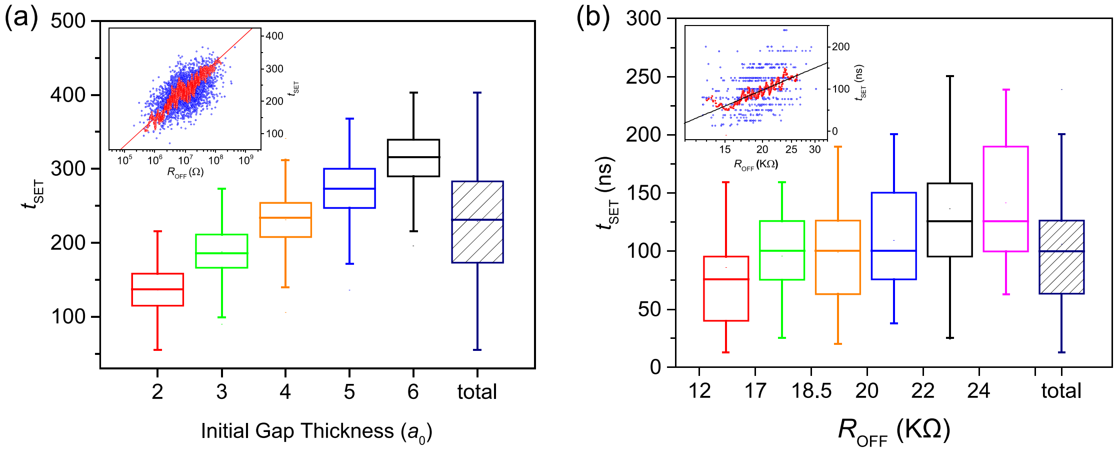

Figure S10 | **Comparison of statistical results of t_{SET} distribution under CVS in **(a) MC simulation (Figure 4d in the main text) and **(b)** previous experiment.[s6]

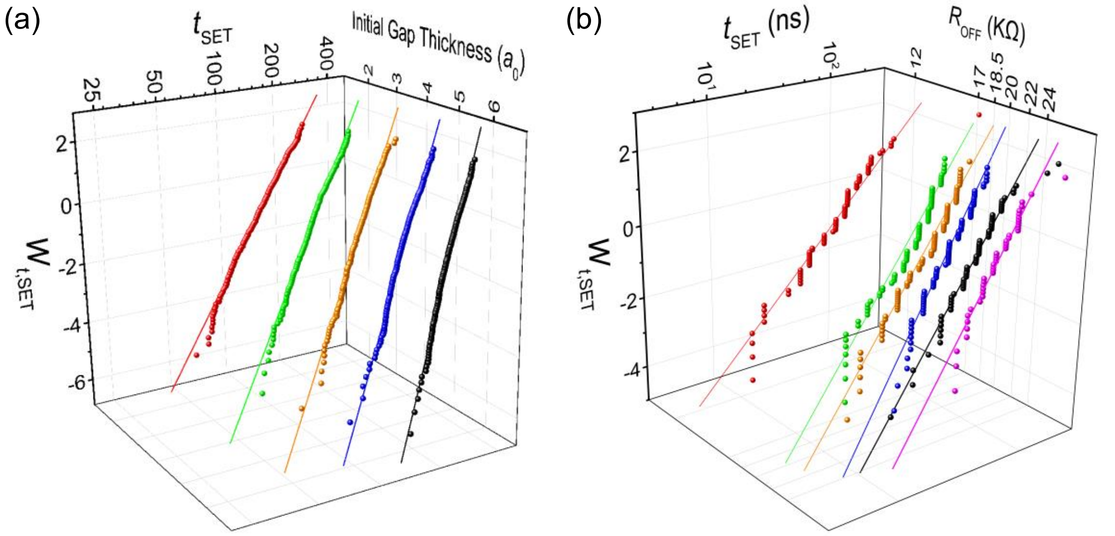

Figure S11 | **Comparison of Weibull distribution of t_{SET} under CVS in **(a) MC simulation (Figure 4e in the main text) and **(b)** previous experiment.[s6]

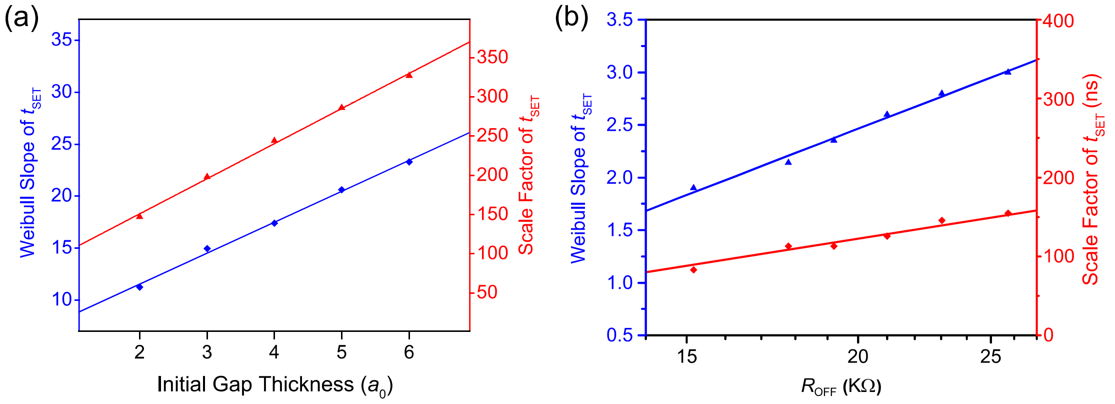

Figure S12 | **Comparison of Weibull slope and scale factor extracted from t_{SET} distribution under VSM in **(a) MC simulation (Figure 4f in the main text) and **(b)** previous experiment.[s6]

2.2.3. Gate Voltage Ramp Programming Operation

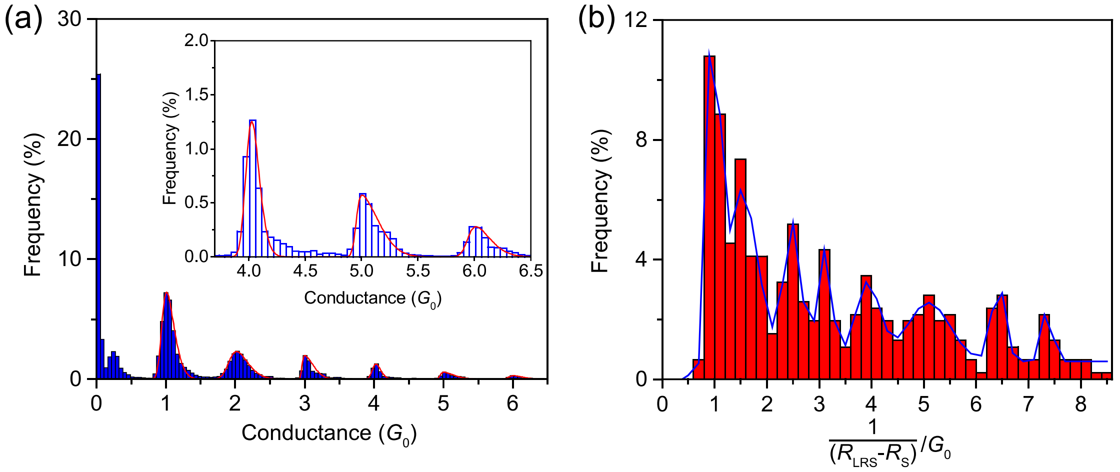

Figure S13 | Comparison of statistical distribution of conductance under GVR in **(a)** MC simulation (Figure 4h in the main text) and **(b)** previous experiment.[s5]

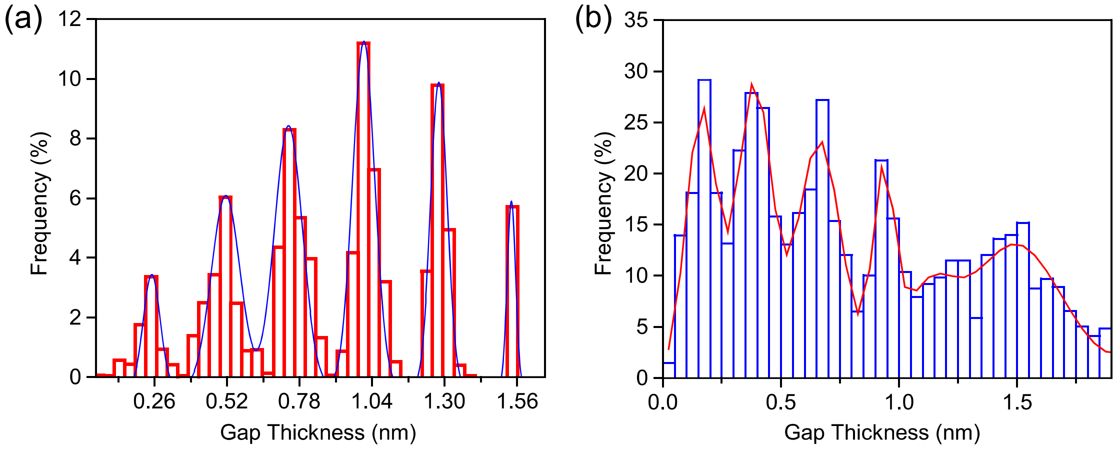

Figure S14 | Comparison of statistical distribution of gap thickness under GVR in **(a)** MC simulation (Figure 4i in the main text) and **(b)** previous experiment.[s5]

3. Evolution of the filament (spatial distribution of conducting/insulating cells) during the SET transition under different electrical stress conditions

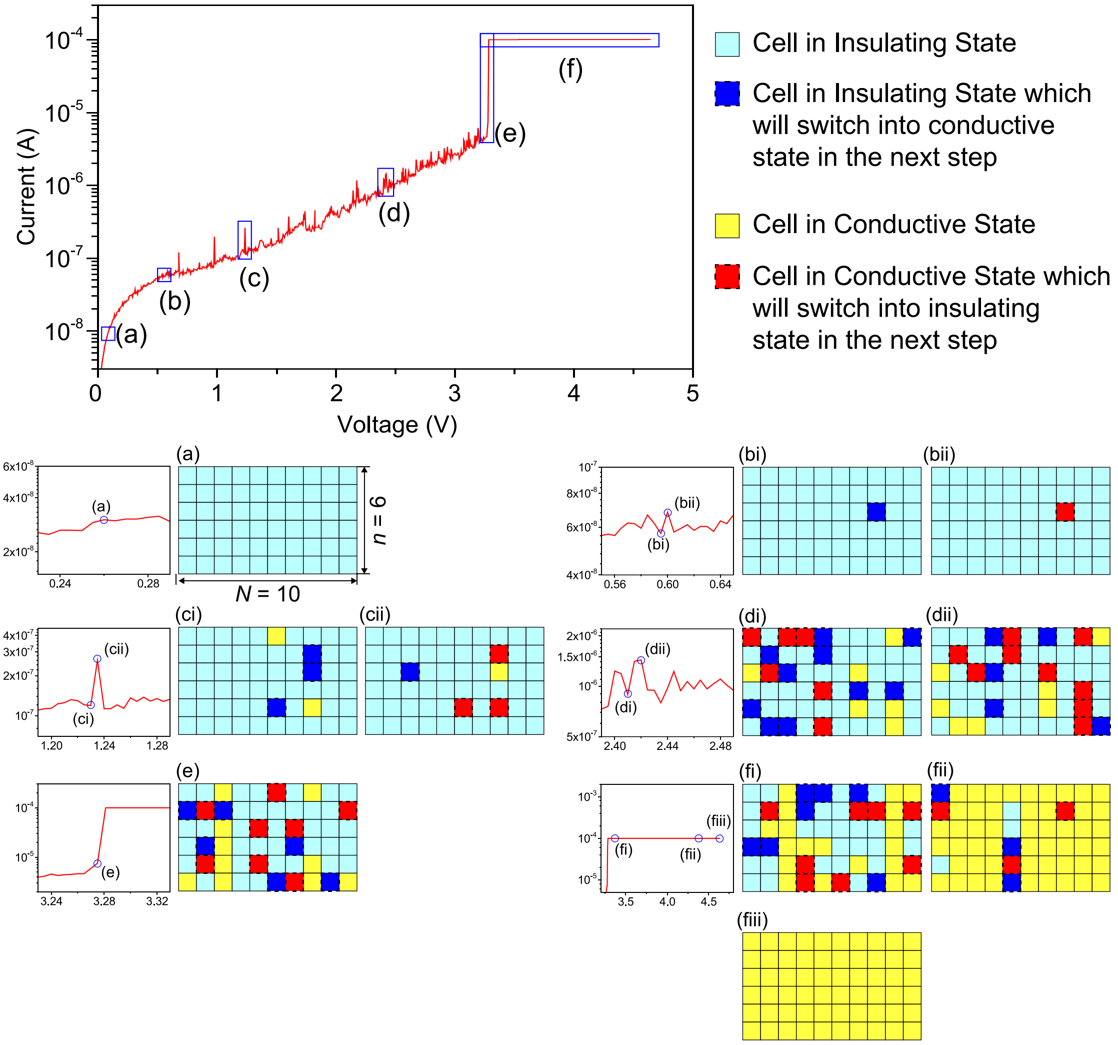

Figure S15 | Color diagrams showing the switching of some of the $n \times N$ cells representing the filament in different stages of a VSM simulated experiment. Dynamic switching is associated to features in the corresponding $I - V$ characteristics.

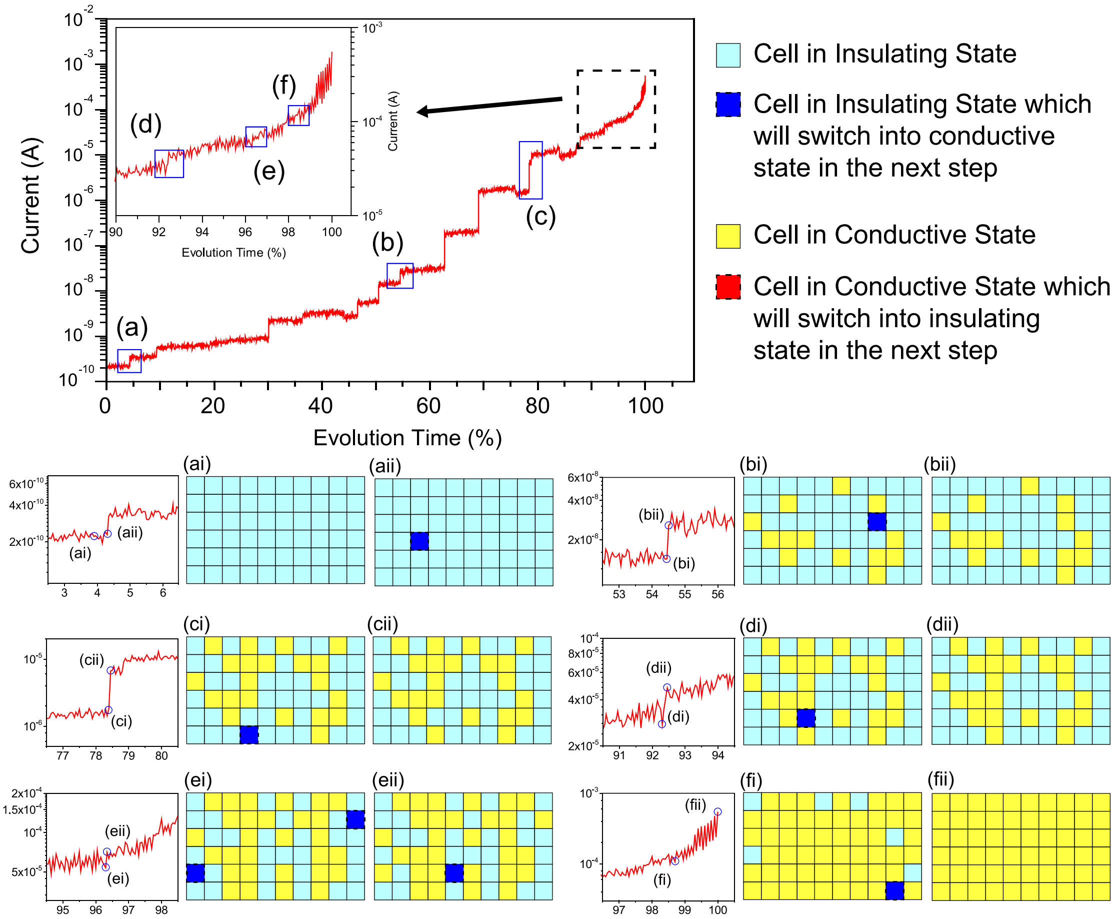

Figure S16 | Color diagrams showing the switching of some of the $n \times N$ cells representing the filament in different stages of a GVR simulated experiment. Dynamic switching is associated to features in the corresponding $I - t$ characteristics.

Reference

S1. Simmons, J. G. Generalized formula for the electric tunnel effect between similar electrodes separated by a thin insulating film. *J. Appl. Phys.* **34,** 1793–1803 (1963).

S2. Long, S. *et al.* A model for the set statistics of RRAM inspired in the percolation model of oxide breakdown. *IEEE Electron Device Lett.* **34,** 999–1001 (2013).

S3. Zhang, M. *et al.* Current compliance impact on the variability of Hf02-based RRAM devices. *IEEE Silicon Nanoelectron. Work. (SNW)* Honolulu, HI 96-97 (2016).

S4. Xu, X. *et al.* Methodology for stability evaluation on the Multi-level storages of oxide-based conductive bridge RAM (CBRAM). *IEEE 22nd Int. Symp. Phys. Fail. Anal. Integr. Circuits (IPFA)* Hsinchu 543-547 (2015).

S5. Lv, H. *et al.* Atomic view of filament growth in electrochemical memristive elements. *Sci. Rep.* **5,** 13311 (2015).

S6. Zhang, M. *et al.* A physical model for the statistics of the Set switching time of resistive RAM measured with the Width-Adjusting Pulse Operation method. *IEEE Electron Device Lett.* **36,** 1303–1306 (2015).
